# Effect of pH on the Mechanical Properties of Single-Biopolymer Mucilage (*Opuntia ficus-indica*), Pectin and Alginate Films: Development and Mechanical Characterisation

**DOI:** 10.3390/polym15244640

**Published:** 2023-12-07

**Authors:** Brandon Van Rooyen, Maryna De Wit, Gernot Osthoff, Johan Van Niekerk, Arno Hugo

**Affiliations:** 1 Department of Sustainable Food Systems and Development, University of the Free State, Bloemfontein 9301, South Africa; 2 Department of Microbiology and Biochemistry, University of the Free State, Bloemfontein 9301, South Africa; 3 Department of Animal Science, University of the Free State, Bloemfontein 9301, South Africa

**Keywords:** packaging, cactus pear mucilage, *Opuntia ficus-indica*, pectin, alginate, biopolymer films, cross-linked, mechanical properties, pH, polymer concentration

## Abstract

Pectin and alginate are well-established biopolymers used in natural film development. Single-polymer mucilage films were developed from freeze-dried native mucilage powder of two cultivars, ‘Algerian’ and ‘Morado’, and the films’ mechanical properties were compared to single-polymer pectin and alginate films developed from commercially available pectin and alginate powders. The casting method prepared films forming solutions at 2.5%, 5%, and 7.5% (*w*/*w*) for each polymer. Considerable variations were observed in the films’ strength and elasticity between the various films at different polymer concentrations. Although mucilage films could be produced at 5% (*w*/*w*), both cultivars could not produce films with a tensile strength (TS) greater than 1 MPa. Mucilage films, however, displayed > 20% elongation at break (*%E*) values, being noticeably more elastic than the pectin and alginate films. The mechanical properties of the various films were further modified by varying the pH of the film-forming solution. The various films showed increased TS and puncture force (PF) values, although these increases were more noticeable for pectin and alginate than mucilage films. Although single-polymer mucilage films exhibit the potential to be used in developing natural packaging, pectin and alginate films possess more suitable mechanical attributes.

## 1. Introduction

The development of biopolymer films, making use of naturally biodegradable materials, has been gaining attention in the food industry. The ever-increasing environmental and human health concerns around the use of petroleum-based plastic packaging have been a driving force surrounding recent developments in this field [1]. Specifically, functional properties associated with the pectin and alginate polysaccharides have been explored in the formulation of natural packaging [2,3]. Pectin and alginate are considered anionic polymers, displaying varying degrees of charge. The chemical structures of pectin and alginate are well-documented and accepted, with their structures directly influencing their functionality. Structural differences between pectin and alginate films have resulted in the different polymer films displaying variations between their mechanical properties, specifically relating to their film strength and elasticity [4,5,6,7,8]. In order to provide adequate protection for food, evaluating the mechanical properties of films is considered essential to ensure the structural integrity of potential food packaging. The mechanical properties of biopolymer films have further been shown to be influenced by several factors, ultimately determining their applications [4,9].

Polymer concentration, changes in pH, and the addition of cross-linking agents have specifically been shown to influence biopolymer films’ physical and mechanical properties. Additionally, film preparation methods and the addition of plasticizers also play a role in determining the specific physical properties displayed by these films. Adding glycerol or sorbitol as a plasticizer can be considered essential to biopolymer film development to ensure films presented by uniform microstructures exhibit desirable physical properties [10,11,12,13].

Although pectin and alginate polymers are well-known for their film-forming abilities, which meet multiple food packaging requirements, the continuous need for natural packaging possessing favorable economic and environmental benefits has promoted the sourcing and investigation of alternative biomaterials. Of specific interest are biopolymers produced in large surplus quantities, which are easily accessible and have relatively low-cost implications, such as cactus pear mucilage precipitate from the cladodes of *Opuntia ficus-indica* [11,14,15]. The functional potential of native mucilage precipitate has allowed for its investigation in developing biopolymer films, although limited research is available thereon [16,17,18].

While native mucilage precipitate has been well-investigated from a molecular level, diversity in its composition has been reported. Generally, cactus pear mucilage is considered a highly flexible heteropolysaccharide with a high molecular weight. Multiple researchers have studied the chemical composition of *Opuntia* spp. mucilage precipitates, often reporting considerable variations thereof [19,20,21]. Although variations in sugar composition and consequential molecular weight have been reported, mucilage molecules are considered to be quite large. This long-chain polymer is represented by varying proportions of D-galacturonic acid and neutral sugars of L-arabinose, D-galactose, L-rhamnose, and D-xylose. The primary mucilage structure is said to be comprised of a linear core chain of repeating (1→4) D-galacturonic acid and (1→2) L-rhamnose [20,21].

Additionally, the mucilage polymer is associated with many side chains of D-galactose attached to the L-rhamnose residues. These complex peripheral chains have shown the potential to be composed of various types of sugars. The galactose side chains can further branch into either arabinose or xylose. Native mucilage chemical composition can vary considerably, depending on the efficacy and type of extraction method used [20,21,22,23,24].

Different fractions present within the native mucilage precipitate have been further investigated. Generally, a charged, pectin-like fraction and a neutral fraction, considered the purer mucilage fraction, represent native mucilage precipitate. Multiple similarities are observed regarding the chemical composition between these two fractions [21]. In general, these two fractions of native mucilage extract display similar sugar composition. However, there are differences concerning the percentages of the sugars between the pectin-like and pure mucilage fractions associated with whole cladodes precipitate of *Opuntia* spp. [21].

The presence of charged sugars in native mucilage is responsible for its structuring capabilities, granted that specific cross-linking parameters are met, behaving similarly to that of pectin and alginate [25]. The presence of charged sugars also allows for the mucilage molecule to be influenced by changes in solution pH. Consequently, changes in solution pH have been shown to alter the functional properties of a native mucilage solution, ultimately influencing its structuring capabilities [16,26].

As with pectin and alginate biopolymer films, a plasticizer is strongly recommended to develop mucilage biopolymer films. ‘Dried’ mucilage films generally displayed brittle and fragile properties if no plasticizer was used in their development [12,27,28].

Recent work published by Van Rooyen et al. [13,29] showed mucilage precipitates’ potential to be used in the development of single-polymer and blended biopolymer films. However, factors such as polymer concentration and the influence that pH would have on single-polymer mucilage films still remain relatively unexplored compared to other well-established biopolymer films, such as biopolymer pectin and alginate films. Van Rooyen et al. [13] specifically reported mucilage precipitate, in some instances, to enhance certain important pectin films’ mechanical properties when added at concentrations lower than that of the pectin-base polymer used. For native mucilage precipitate to be considered a possible viable, novel, and natural packaging alternative to pectin and alginate, it is essential to better understand its homopolymeric (single-polymer) film-forming potential and influencing factors, such as pH. Therefore, this study aimed to determine the mechanical properties of different *Opuntia ficus-indica* cultivars’ native mucilage single-biopolymer (homopolymeric) films to similarly developed pectin and alginate single-biopolymer films while considering the influence of polymer concentration and pH.

## 2. Materials and Methods

### 2.1. Materials

#### 2.1.1. Commercial Film-Forming Components

Pectin powder from apples (Sigma-Aldrich, Cape Town, South Africa) represented by a 50–75% degree of esterification and a ≤10% moisture content, sodium alginate powder (Sigma-Aldrich, Cape Town, South Africa) with both glucuronic and mannuronic acid content, glycerol, and >99% purity (Merck, Johannesburg, South Africa) was used.

#### 2.1.2. pH Regulators

The pH was adjusted with either acetic acid glacial (100%) or sodium hydroxide (98%) (Merck, Johannesburg, South Africa) were used.

#### 2.1.3. Mucilage Precipitation and Freeze-Drying of Precipitate into Powder

Following a patented method by Du Toit and De Wit [30], as described by Van Rooyen et al., 2023, native mucilage precipitations were prepared from the mature cladodes (~24 months old) of ‘Algerian’ and ‘Morado’ cultivars of *Opuntia ficus-indica*. Cladodes were sourced from a well-established cactus pear orchard at the University of the Free State, South Africa, with a cactus pear plant density of 666 cactus pear/ha, unirrigated. This specific, patented method is well-suited for this work due to its cost-effectiveness and repeatability. Furthermore, the resultant precipitate has been well-characterized in previous studies conducted [31,32]. A simplified version of this procedure describes the entire, unpeeled cladode being sliced into approximately 2–3 cm cubes and microwaved on high (900 w) for a time of 4 min at 900 W, followed by maceration of the cubes in a pulp. The pulp was then more easily centrifuged (Beckman^®^ Centrifuge 2315, Brea, CA, USA) to separate the mucilage precipitate effectively from the solids. The pulp was specifically centrifuged at 8000 rpm for 15 min at 8000 rpm at a constant temperature of 4 °C. The freeze-drying process of the mucilage precipitate to form the mucilage powder used in this current work required moisture removal from the liquid precipitate until a 95% weight loss was achieved. Using a mortar and pestle, samples were then milled into a consistent, fine powder. All freeze-drying required that samples were kept under constant vacuum and low temperatures of −30 °C to −40 °C.

### 2.2. Preparation of Various Film-Forming Solutions

With some modifications, standardized approaches were used to form all film-forming solutions prepared, as described by Van Rooyen et al. [29]. Film-forming solutions were prepared by manually dispersing the required amounts of polymer into distilled water. Distilled water used to prepare all film-forming solutions required the addition of glycerol. Glycerol additions were added at 20% (*w*/*w*, based on the weight of the polymer used to develop the films) to the mucilage film-forming solutions and 60% for pectin and alginate film-forming solutions. A minimum of 30 min was allowed for all dispersed polymer solution rehydration at room temperature (~25 °C) under constant stirring (Freed Electric-Model MH-4, Rehovot, Israel). Ensuring homogeneity, all solutions were mechanically mixed for 10 s (Mellerwave Stick Blender-Model 85200, Cape Town, South Africa), and potential entrapped air was removed from the solutions using a Genesis vacuum sealer (Verimark (Pty) Ltd., Pretoria, South Africa) [13,29].

### 2.3. Single-Polymer Film Development

A standardized film casting method was used to develop the films used in this study, with some modifications, as described by Van Rooyen et al. [29]. The casting method required 70 g of prepared film-forming solution to be evenly spread into 140 mm Petri dishes, forming a film that once dried could be removed for further evaluation [29]. In this current work, films were dried at 40 °C for 24 h using an EcoTherm ventilated oven (Model 920, 1000 W, Labotec, Johannesburg, South Africa). Films were equilibrated for 24 h at room temperature (~25 °C) in a closed container before evaluation.

### 2.4. pH Adjustment

The process of pH adjustment involved altering the pH of the homogenous film-forming solution by adding either 1 M sodium hydroxide or 1 M acetic acid until the desired pH ranges of pH 9–10 and pH 3–3.5 were achieved.

### 2.5. Evaluation of Film Mechanical Properties

Making use of a Brookfield AMETEK^®^ CT3™ Texture Analyzer (Westville, South Africa), both tensile and puncture tests were completed on a total of 12 conditioned films cut into 25 × 50 mm strips per treatment. The ASTM International standard methods (ASTM-D882:2010) [33] were followed, similar to that described by Van Rooyen et al. [29], for all mechanical tests completed. The maximum stress (force/area) a film can withstand before it breaks when an external force is applied to the film is considered its tensile strength, with film thickness determined using a digital micrometer (Grip, Johannesburg, South Africa) [4,29]. Elongation at break measures a film’s potential to extend until the point where it ruptures [4]. The tensile strength and elongation at break percentages of films are represented by the maximum load divided by the cross-sectional areas of the initial film and the change in the initial length of the films at rupture, respectively [29]. Film tensile tests were approached using the texture analyzer fitted with the Brookfield AMETEK^®^ TA-RCA Roller Cam Accessory grips. A test speed of 0.80 mm·s^−1^, grip spacing of 50 mm, and test distance of 35 mm were selected.

#### Puncture Test Evaluation

Using a 4 mm Brookfield AMETEK^®^ TA44 probe and accompanying texture analyzer, puncture tests (puncture force and distance to puncture) were completed for the various films. A test speed of 0.80 mm·s^−1^ was selected for this work, similar to that selected in recent studies by Van Rooyen et al. [13,29]. The puncture force and distance to puncture values were determined by a software-generated force-distance graph using the texture analyzers’ accompanying software.

### 2.6. Experimental Design

In the first part of this study, mucilage, pectin, and alginate films were developed at 2.5%, 5%, and 7.5% concentrations at their respective native (unchanged) pH with the addition of glycerol as a plasticizer to determine the effect polymer concentration had on the different film mechanical properties.

Secondly, mucilage, pectin, and alginate film-forming solutions, prepared at 5% (*w*/*w*) together with the prescribed glycerol inclusions, were all subjected to pH alterations at both pH 3–3.5 and pH 9–10 using either acetic acid or sodium hydroxide. Thereafter, the different pH-adjusted film-forming solutions were cast, dried, and tested.

### 2.7. Statistical Analysis

Upon completion of the various trials, the data were captured using Microsoft Excel (2016) and subjected to analysis of varience statistical analysis (ANOVA). Specifically, a one-way analysis of variance (NCSS Statistical Software package, version 11.0.20) was completed on the data. Using the Tukey–Kramer multiple comparison test (α = 0.05), significant differences were identified between the treatment means (NCSS Statistical Software package —v11.0.20, Salt Lake City, UT, USA).

## 3. Results

### 3.1. Film Concentration

#### 3.1.1. Film Thickness

**Figure 1:**
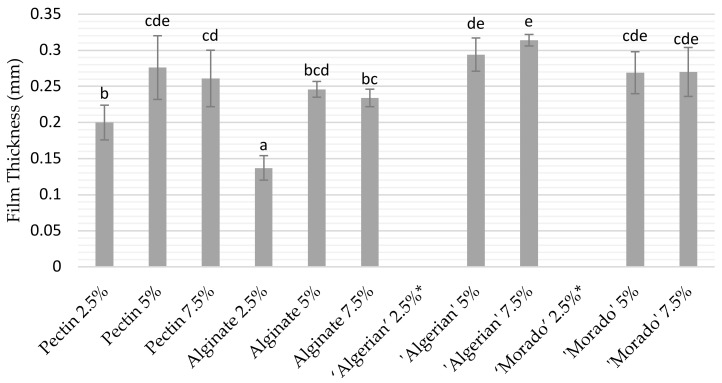
Film thickness of pectin and alginate, as well as, ‘Algerian’ and ‘Morado’ mucilage films at concentrations of 2.5%, 5%, and 7.5%. The mean values of the different treatments, together with their standard deviation error bars, are displayed. Error bars with different superscripts differ significantly (*p* < 0.05). * Below minimum measurable film thickness.

Not all tested concentrations allowed for the successful formation of mucilage films at their native (unchanged) pH, as both the ‘Algerian’ and ‘Morado’ mucilage could not successfully form films at a concentration of 2.5%. Increasing the mucilage concentration to 5% and 7.5% allowed the successful formation of both ‘Algerian’ and ‘Morado’ films. Pectin and alginate formed films at 2.5%, 5%, and 7.5% polymer concentrations. Pectin and alginate film thickness significantly increased by increasing the concentration from 2.5% to 5% (*p* < 0.05). Concentration did not significantly influence the film thickness when increasing the concentration from 5% to 7.5% for the mucilage, pectin, and alginate films (*p* > 0.05) (Figure 1).

The influence of varying the polymer concentrations on the tensile and puncture tests of mucilage, pectin, and alginate films was considered, as presented in Table 1 and Table 2.

#### 3.1.2. Tensile Tests

Differences in the mechanical properties between the various polymer films at increasing concentrations were observed.

Increasing the polymer concentration from 2.5% to 5% significantly increased film tensile strength (*p* < 0.05) (Table 1). However, increasing the polymer concentration from 5% to 7.5% did not significantly influence the tensile strength (TS) of mucilage, pectin, and alginate films (*p* > 0.05). Alginate films showed significantly higher TS values than their pectin film counterparts (*p* < 0.05). Galus and Lenart [7] also reported alginate films to have a greater TS than pectin films. These differences were accounted for by polymer variation, with pectin displaying a less organized polymer network than alginate [7]. ‘Algerian’ and ‘Morado’ films showed significantly poorer TS than pectin and alginate films, irrespective of the concentration (*p* < 0.05). Gheribi et al. [17] also showed that 4% (*w*/*w*) films produced from purified *Opuntia ficus-indica* mucilage, with the addition of glycerol as a plasticizer, were represented by low TS values of ±1 MPa. Espino-Díaz et al. [16] also reported mucilage films to have TS values similar to those reported in the current research, with low TS values accounting for the mucilage films’ high molecular weight distribution. Differences observed between the TS of different polymer films have been attributed to differences in polymer molecular weight and inter- and intramolecular association between polymer chains [16,34].

In addition to the film TS, film elongation at break percentage (*%E*) is also considered essential when evaluating a film’s mechanical properties, as an adequate film needs to display both resistance and flexibility [7,17]. Increasing the polymer concentration showed trends in increasing the *%E* values for the various polymer films. At a 7.5% polymer concentration, all mucilage films showed significantly better (*p* < 0.05) *%E* values when compared to pectin and alginate polymer films. Interestingly, mucilage films showed higher *%E* values but relatively low TS compared to pectin and alginate films. Espino-Díaz et al. [16] also investigated mucilage films with low TS values, which showed excellent elasticity.

#### 3.1.3. Puncture Tests

The puncture tests were used to evaluate the mechanical properties of the various polymer films (mucilage, pectin, and alginate) at increasing polymer concentrations (Table 2).

Considering the puncture force (PF) values, increasing the polymer concentration of the various films tested resulted in an increase in the force required to puncture the films (Table 2). Pectin and alginate films showed significantly greater PF values than ‘Algerian’ and ‘Morado’ mucilage films at all concentrations tested (*p* < 0.05). Similar findings were reported for the mucilage films when evaluating their tensile strength, confirming mucilage films to have inferior mechanical strength compared to the pectin and alginate films, regardless of the different polymer concentrations tested in this current work. Sandoval et al. [14] suggested that low puncture force resistance values for mucilage films would be influenced by concentration, with low mucilage concentrations being associated with decreased film strength. Unlike the tensile data, the puncture test data suggested that the mucilage films possessed no clear superior elastic properties compared to the pectin and alginate films. In fact, at 7.5% concentration, alginate films showed significantly greater distance to puncture (DTP) values than any of the other films investigated (*p* < 0.05) (Table 2).

### 3.2. Film pH

#### 3.2.1. Film Thickness and the Influence of pH

**Figure 2:**
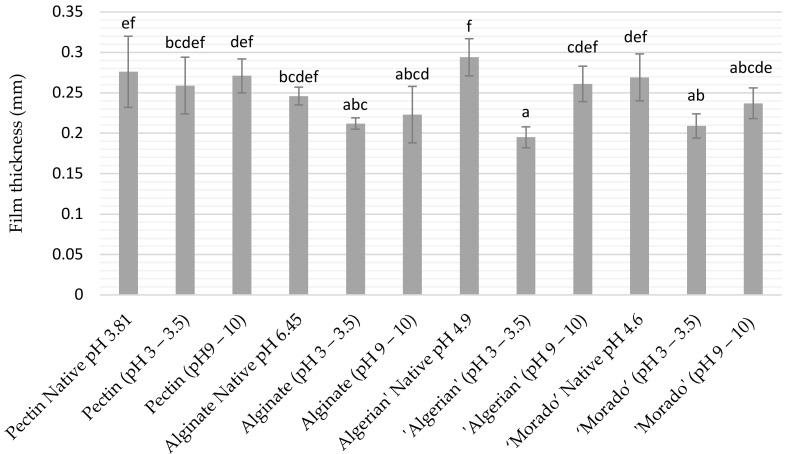
Film thickness of pectin and alginate films, as well as, ‘Algerian’ and ‘Morado’ mucilage films at varying pH. The mean values of 12 different treatments are displayed together with their standard deviation error bars. Error bars represented by different superscripts differed significantly (*p* < 0.05).

Changes in pH showed little influence on the pectin and alginate films’ thickness compared to their native pH films (*p* > 0.05). These trends were not observed for mucilage films, as both ‘Algerian’ and ‘Morado’ film thickness significantly decreased (*p* < 0.05) at a lower pH, with only minimal changes in film thickness reported at pH 9–10 in comparison to their native pH counterpart films (Figure 2).

#### 3.2.2. pH and Film Tensile Measurements

Pectin and alginate films were shown to be significantly influenced by a decrease in pH (*p* < 0.05), resulting in an increase in the film’s TS values, which was not observed for mucilage films (Table 3). Only pectin films showed a significant increase in TS values (*p* < 0.05) compared to their native pH counterpart films when the films’ pH was increased.

The films’ *%E* values were also considered. It was observed that both ‘Algerian’ and ‘Morado’ mucilage produced films displaying the highest *%E* values at all pH ranges tested. Specifically, decreasing the film pH resulted in significant increases (*p* < 0.05) in film *%E* values compared to their counterpart films at native pH. Espino-Díaz et al. [16] also reported trends of increases in a mucilage film *%E* when the pH of the film was decreased.

#### 3.2.3. pH and Film Puncture Tests

Considering the various films’ PF values, decreasing the pH of mucilage, pectin, and alginate films showed trends of increased PF values compared to their counterpart films at native pH (Table 4). However, only the pectin and alginate films’ PF values were significantly increased (*p* < 0.05) when decreasing the film pH (pH 3–3.5). Lastly, the DTP values of only ‘Algerian’ mucilage films at pH 9–10 were significantly increased (*p* < 0.05) compared to ‘Algerian’ films at native pH. ‘Morado’ mucilage films’ DTP values were not significantly influenced (*p* > 0.05) by changes in pH (Table 4).

## 4. Discussion

The mechanical properties of biopolymer films are fundamental as these parameters directly evaluate the physical integrity expected from potential food packaging. Biopolymer films envisaged to be used for packaging need to provide strength and elasticity [17,34]. The tensile tests (tensile strength and elongation at break percentage) and puncture tests (puncture force and distance to puncture) were used to evaluate the film strength and elasticity. Overall, increasing the polymer concentration in mucilage, alginate, and pectin films directly influenced film strength and elasticity. Furthermore, the mechanical properties of the various polymer films investigated were influenced differently by increasing the polymer concentration in the films. Increasing the mucilage concentration for both ‘Algerian’ and ‘Morado’ films enhanced both film strength and elasticity, potentially due to the increased likeness of polymer association and entanglement, which can be expected when increasing a polymer concentration in film development. Although mucilage films showed lower film strength when compared to commercially available pectin and alginate films, mucilage films still displayed adequate mechanical properties, specifically displaying excellent elasticity.

At all pH ranges tested, pectin and alginate films showed superior film strength, as they reported higher tensile strength (TS) and puncture force (PF) values when compared to the mucilage films. Furthermore, pectin and alginate films showed that pH considerably influenced the resultant film strength. Regardless of the pH range tested, only minimal changes were reported for the various mucilage film strengths (Table 3 and Table 4). At a pH of 3–3.5, pectin and alginate film TS and PF values were significantly increased (*p* < 0.05) compared to their counterparts at their native pH. The literature has suggested that certain charged polymers have the ability to undergo a type of acid gelation, forming structured hydrogels by increasing polymer chain association [35]. Although minimal, trends of increased film strength were reported for both ‘Algerian’ and ‘Morado’ films at pH 3–3.5, compared to their counterparts at native pH. It is thus postulated that the mucilage used in developing these biopolymer films has the potential to undergo a type of acid gelation due to the increased film strength observed at a decreased pH. These findings were further supported by a decrease in mucilage film thicknesses at pH 3–3.5, as films that display decreased thickness might be linked to an increased polymer organization within the film matrix. Consequently, increased polymer organization would result in less intermolecular spacing between the polymer chains, which is strongly associated with the gelation of charged polymers [5].

Similarly, due to a type of acid gelation, pectin films displayed increased film strength at a decreased pH as there was an expected reduction in electrostatic repulsion between their polymer chains, resulting in a consequent association of their polymer chains [36]. Furthermore, acid gelation is responsible for developing a more organized polymer network, as in the case of alginate films, as a more organized polymeric structure would allow for increased cohesion forces between the polymer chains, ultimately responsible for enhancing film mechanical properties [4,5]. Although both pectin and alginate polymers are able to undergo acid gelation, differences in their mechanical properties, specifically regarding elasticity, highlight differences between the two polymer film-forming abilities. Mucilage films generally showed superior *%E* values. However, when evaluating their DTP values, little elastic advantage was observed for the mucilage films compared to the pectin and alginate films.

## 5. Conclusions

Cactus pear mucilage was successfully used to develop biopolymer films at >5% concentrations. Decreasing the pH of the different biopolymer films influenced their mechanical properties differently. Pectin and alginate films reported increased tensile strength and puncture force values at a decreased pH, while mucilage film strength was only minimally influenced by changes in pH.

It is postulated that the acid gelation of the pectin and alginate films was responsible for these increases in film strength observed in this current work. Although no significant differences (*p* > 0.05) were reported for mucilage films at a decreased pH, in comparison to the mucilage films at native pH, trends of increased film strength were observed. As the pectin and alginate films’ mechanical properties showed a greater dependency on pH than mucilage films, the native mucilage precipitate was therefore believed to be presented by a less charged polymer than the pectin and alginate polymers used in this current work.

Overall, mucilage was successfully used in developing biopolymer films, highlighting its potential to be used in developing natural packaging. Mucilage films were, however, much weaker overall than pectin and alginate films, although they displayed excellent elasticity. Mucilage shows some commercial viability when comparing its mechanical properties to those of pectin and alginate. It is, however, recommended that future research should investigate additional film properties, specifically the water and gas barrier properties of these films. However, mucilage films are not likely to find similar packaging applications as expected from alginate and pectin films.

## Figures and Tables

**Table 1:** The tensile tests of mucilage, pectin, and alginate films at increasing polymer concentrations.

Treatments	Tensile Strength (MPa)	Elongation at Break %
Pectin 2.5%	3.31 ± 0.43 ^b^	5.59 ± 1.51 ^a^
Pectin 5%	6.41 ± 0.50 ^c^	14.32 ± 1.88 ^bc^
Pectin 7.5%	6.27 ± 1.11 ^c^	18.98 ± 5.66 ^cd^
Alginate 2.5%	10.20 ± 1.38 ^d^	4.91 ± 0.75 ^a^
Alginate 5%	17.57 ± 0.90 ^e^	7.79 ± 1.03 ^a^
Alginate 7.5%	16.88 ± 0.69 ^e^	21.99 ± 0.69 ^d^
‘Algerian’ 2.5% *		
‘Algerian’ 5%	0.26 + 0.05 ^a^	33.10 ± 6.10 ^e^
‘Algerian’ 7.5%	1.17 ± 0.08 ^a^	49.82 ± 1.53 ^f^
‘Morado’ 2.5% *		
‘Morado’ 5%	0.32 ± 0.10 ^a^	21.58 ± 1.80 ^d^
‘Morado’ 7.5%	1.36 ± 0.15 ^a^	47.79 ± 5.84 ^f^
Significance level	*p* < 0.005	*p* < 0.005

* Below minimum measurable film tensile mechanical properties. Where applicable, the mean values of 12 different treatments are displayed together with their standard deviations (±). The mean values represented by different superscripts in the same column differed significantly (*p* < 0.05).

**Table 2:** Puncture tests of mucilage, pectin, and alginate films at increasing polymer concentrations.

Treatments	Puncture Force (N)	Distance to Puncture (mm)
Pectin 2.5%	25.67 ± 5.51 ^b^	3.51 ± 0.78 ^ab^
Pectin 5%	31.75 ± 2.38 ^bc^	4.04 ± 0.38 ^abc^
Pectin 7.5%	47.85 ± 4.17 ^d^	5.98 ± 0.62 ^e^
Alginate 2.5%	34.47 ± 2.72 ^c^	4.02 ± 0.23 ^abc^
Alginate 5%	72.17 ± 4.68 ^e^	5.61 ± 0.37 ^de^
Alginate 7.5%	72.22 ± 8.23 ^e^	8.63 ± 0.75 ^f^
‘Algerian’ 2.5% *		
‘Algerian’ 5%	2.43 + 0.26 ^a^	4.24 ± 0.63 ^abc^
‘Algerian’ 7.5%	5.67 ± 0.64 ^a^	3.09 ± 0.93 ^a^
‘Morado’ 2.5% *		
‘Morado’ 5%	1.82 ± 0.21 ^a^	4.71 + 0.89 ^bcd^
‘Morado’ 7.5%	8.30 ± 0.70 ^a^	4.99 ± 0.45 ^cde^
Significance level	*p* < 0.005	*p* < 0.005

* Below minimum measurable film puncture mechanical properties. Where applicable, the mean values of 12 different treatments are displayed together with their standard deviations (±). The mean values represented by different superscripts in the same column differed significantly (*p* < 0.05).

**Table 3:** The mechanical properties of mucilage, pectin, and alginate biopolymer films at their native pH and at pH 3–3.5 and pH 9–10.

Treatments/Films	Tensile Strength (MPa)	Elongation at Break %
Pectin Native pH 3.81	6.41 ± 0.50 ^b^	14.31 ± 1.88 ^b^
Pectin (pH 3–3.5)	11.52 ± 1.06 ^d^	2.39 ± 0.34 ^a^
Pectin (pH9–10)	8.51 ± 1.30 ^c^	4.68 ± 1.04 ^a^
Alginate Native pH 6.45	17.57 ± 0.90 ^e^	7.79 ± 1.03 ^a^
Alginate (pH 3–3.5)	20.71 ± 0.85 ^f^	5.38 ± 0.23 ^a^
Alginate (pH 9–10)	17.58 ± 0.73 ^e^	5.17 ± 0.48 ^a^
‘Algerian’ Native pH 4.9	0.262 ± 0.05 ^a^	33.10 ± 6.10 ^d^
‘Algerian’ (pH 3–3.5)	1.01 ± 0.15 ^a^	55.01 ± 6.32 ^e^
‘Algerian’ (pH 9–10)	0.27 ± 0.09 ^a^	14.19 ± 0.61 ^b^
‘Morado’ Native pH 4.6	0.31 ± 0.10 ^a^	21.58 ± 1.76 ^c^
‘Morado’ (pH 3–3.5)	0.71 ± 0.10 ^a^	36.53 ± 3.69 ^d^
‘Morado’ (pH 9–10)	0.48 ± 0.03 ^a^	35.42 ± 5.13 ^d^
Significance level	*p* < 0.005	*p* < 0.005

The mean values of 12 different treatments are displayed together with their standard deviations (±). The mean values represented by different superscripts in the same column differ significantly (*p* < 0.05).

**Table 4:** Puncture tests of pH-altered mucilage, pectin, and alginate films at their native pH, pH 3–3.5, and pH 9–10.

Treatments/Films	Puncture Force (N)	Distance to Puncture (mm)
Pectin Native pH 3.81	31.75 ± 2.38 ^b^	4.04 ± 0.38 ^bc^
Pectin (pH 3–3.5)	44.19 ± 4.32 ^c^	2.68 ± 0.48 ^a^
Pectin (pH9–10)	41.95 ± 8.77 ^c^	3.77 ± 0.83 ^b^
Alginate Native pH 6.45	72.17 ± 4.68 ^d^	5.61 + 0.37 ^de^
Alginate (pH 3–3.5)	80.29 ± 2.12 ^e^	5.00 ± 0.41 ^cde^
Alginate (pH 9–10)	73.78 ± 4.54 ^de^	5.89 ± 0.49 ^e^
‘Algerian’ Native pH 4.9	2.43 ± 0.26 ^a^	4.24 ± 0.63 ^bc^
‘Algerian’ (pH 3–3.5)	2.65 ± 0.56 ^a^	4.17 ± 0.48 ^bc^
‘Algerian’ (pH 9–10)	2.10 ± 0.67 ^a^	5.99 ± 0.45 ^e^
‘Morado’ Native pH 4.6	1.82 ± 0.21 ^a^	4.71 ± 0.88 ^bcd^
‘Morado’ (pH 3–3.5)	3.43 ± 0.34 ^a^	3.78 ± 0.16 ^b^
‘Morado’ (pH 9–10)	3.15 ± 0.51 ^a^	4.22 ± 0.25 ^bc^
Significance level	*p* < 0.005	*p* < 0.005

The mean values of 12 different treatments are displayed together with their standard deviations (±). The mean values represented by different superscripts in the same column differ significantly (*p* < 0.05).

## Data Availability

Data are contained within the article.
